# Walking Ankle Biomechanics of Individuals With Transtibial Amputations Using a Prescribed Prosthesis and a Portable Bionic Prosthesis Under Myoelectric Control

**DOI:** 10.1109/TNSRE.2024.3440257

**Published:** 2024-08-26

**Authors:** Nicole E. Stafford, Eddie B. Gonzalez, Daniel P. Ferris

**Affiliations:** Department of Mechanical and Aerospace Engineering, University of Florida, Gainesville, FL 32611 USA; Gainesville, FL, USA.; J. Crayton Pruitt Family Department of Biomedical Engineering, University of Florida, Gainesville, FL 32611 USA.

**Keywords:** Myoelectric control, transtibial prosthesis, open source leg

## Abstract

Individuals with transtibial amputation can activate residual limb muscles to volitionally control robotic ankle prostheses for walking and postural control. Most continuous myoelectric ankle prostheses have used a tethered, pneumatic device. The Open Source Leg allows for myoelectric control on an untethered electromechanically actuated ankle. To evaluate continuous proportional myoelectric control on the Open Source Ankle, we recruited five individuals with transtibial amputation. Participants walked over ground with an experimental powered prosthesis and their prescribed passive prosthesis before and after multiple powered device practice sessions. Participants averaged five hours of total walking time. After the final testing session, participants indicated their prosthesis preference via questionnaire. Participants tended to increase peak ankle power after practice (powered 0.80 ± 1.02 W/kg and passive 0.39 ± 0.31 W/kg). Additionally, participants tended to generate greater ankle work with the powered prosthesis compared to their passive device (0.13 ± .15 J/kg increase). Although work and peak power generation were not statistically different between the two prostheses, participants preferred walking with the prosthesis under myoelectric control compared to the passive prosthesis. These results indicate individuals with transtibial amputation learned to walk with an untethered powered prosthesis under continuous myoelectric control. Four out 5 participants generated larger magnitudes in peak power compared to their passive prosthesis after practice sessions. An additional important finding was participants chose to walk with peak ankle powers about half of what the powered prosthesis was capable of based on mechanical testing.

## I. Introduction

Future bionic lower leg prostheses that allow powered assistance under direct control from users could improve mobility and quality of life for individuals with lower limb amputations. Current commercially available lower leg prostheses do not have volitional control strategies that allow users to perform a wide range of movements. Powered ankle prostheses currently available do increase mobility by increased plantar flexion power and providing net positive work to the end of the stance phase [1], [2], [3]. Typically, the controllers modulate joint impedance based on detection of discrete gait phases (i.e. a finite-state machine) [4], [5], [6], [7]. Machine learning algorithms can allow for increased variability and shaping of the finite state machine controller by not completely relying on heuristically determined state switching criteria [8], [9], [10], [11], but they are still limited in flexibility to navigate crowds, obstacles, and time-varying stepping patterns. Finite state machine controllers also do not provide a range of responses to unexpected perturbations at present [12], [13]. Limitations in flexibility are important because lack of controllability and poor mobility are main contributors of prosthesis rejection [14], [15], [16], [17], [18]. Developing prostheses to restore direct, feedforward control of volitional movements would allow individuals with amputations to freely alter their joint mechanics from step to step or for non-stepping movements (e.g., going up on the toes to reach an object on a high shelf).

Biological signals as prosthetic controller inputs could yield intuitive adaptable prosthetic controllers that allow for navigation of complex real-world environments. Lower limb muscle activity captured with electromyography (EMG) can be used to inform current state or movement intentions for prosthetic controllers. Controllers that incorporate EMG signals can be divided into two approaches, supervisory and direct control [19]. Many supervisory controllers rely on machine learning algorithms and electromyography (EMG) and/or mechanical sensors to determine a state classification for a gait event or terrain type. This is similar to other finite state machine controllers that do not use EMG as a signal input. The second main subgroup of EMG controllers is direct control, which uses EMG as a control input to directly modulate prosthesis dynamics [20], [21]. While direct EMG control may increase cognitive load when first learning to walk [22], [23], [24], using a simple intuitive controller could allow users to learn over time to adapt their muscle activity to complete a variety of non-cyclic tasks. Additionally, users report that direct EMG control increases “sense of self” [25], [26], [27], [28], [29], [30] and are preferred over state-based controllers [20]. Another advantage of direct EMG control is that it can incorporate inherent physiological perturbation responses and anticipatory postural control actions into the performance of the prosthesis [13], [22], [23], [31] after multiple sessions with the prosthesis. Most of these laboratory studies have used a tethered pneumatic ankle prosthesis, which does not allow for the evaluation of myoelectric control in real-world environments.

The purpose of this study was to compare gait biomechanics of individuals with transtibial amputation walking with their passive prescribed prosthesis and an experimental bionic prosthesis under proportional myoelectric control. Prior work from our lab examined individuals with lower limb transtibial amputation walking with myoelectric control of an experimental pneumatic bionic prosthesis tethered to an external air supply [22], [32]. Without visual feedback of their prosthesis power, participants walked with a biomechanical gait pattern similar to the pattern they used when walking with their passive prosthesis. Since that publication, a research group at the University of Michigan created an Open Source Leg (OSL) with an electromechanical actuator that is completely portable. It allows for testing of controllers off of treadmills and in environments outside of research laboratories. One group has used the OSL to evaluate direct myoelectric control walking on a treadmill after a 10 minute familiarization period [20]. We used the ankle configuration of the Open Source Leg [33], [34] with proportional myoelectric control to determine how it would affect walking dynamics in individuals with lower limb amputations after multiple familiarization sessions. This initial study was to determine how individuals with transtibial amputation would walk using electromechanical powered prosthesis under continuous myoelectric control. We measured and compared over ground ankle kinematics, kinetics, and leg EMG magnitude and variability as subjects walked in a gait lab after approximately five hours of non-treadmill walking to walking with a prescribed passive prosthesis. We quantified peak ankle power values with the bionic prosthesis under proportional myoelectric control and their passive prosthesis.

## II. Methods

### A. Prosthesis and Controller

We implemented a direct myoelectric controller on the Open Source Leg (OSL) V1 ankle developed at the University of Michigan for this study [33]. The Open Source Leg weighing 2.25 kg has no series elasticity, a range of motion of 30 degrees and was equipped with a 6-axis load cell M3564F (Sunrise Instruments, Shanghai, China). The Open Source Leg provided 10 degrees of dorsiflexion and 20 degrees of plantar flexion. A Raspberry Pi 4 ran the controller in Python at 450 Hz. EMG was collected via the Coapt amplifier (Coapt, LLC Chicago, IL, USA) using Neuroline Surface Electrodes 715 (Ambu, Columbia, MD, USA) which are 1.3 mm thick placed on the residual limb inside the prosthetic socket. Each participant wore a front pack that held the Raspberry Pi, sync generator, batteries (Venom Fly 30 C/11.1V) and EMG system, resulting in a total mass of 1.4 kg.

The controller design was impedance based where muscle activity influenced the set angle. We placed EMG sensors on the residual gastrocnemius and tibialis anterior muscle, however only the gastrocnemius was used for control of the Open Source Leg. At each visit, a maximum voluntary contraction of the participant gastrocnemius was measured and a percentage of their maximum contraction (between 50% - 75%) was used to normalize the EMG signal for prosthetic control. To generate a continuous proportional myoelectric control signal, the residual EMG was processed in real time. The EMG signal was high-pass filtered (second order Butterworth 70 Hz) to attenuate signal artifacts, then the high-passed signal was full wave rectified. Then the full wave rectified signal was low-pass filtered (second order Butterworth 3 Hz). The normalized continuous control signal was proportionally mapped to the impedance set angle parameter with a normalized value of one corresponding to a set angle of 30 degrees. When the participants’ muscle activity was below a baseline threshold, the prosthesis held between a 4 to 6 degree dorsiflexed position. The ankle returned to the baseline position as the muscle activity decreased based on the impedance set angle mapping. A slightly more dorsiflexed position was chosen to provide more natural ankle roll over during walking. Stiffness and damping parameters of the impedance controller were adjusted based on loading of the prosthesis and participant weight to produce less stiffness during beginning and middle of stance. The sagittal moment direction of the onboard load cell was used to determine when the participant began to shift their weight for plantar flexion to increase the stiffness parameters. More information about the controller impedance parameters is provided in the Supplementary Materials S1.

### B. Mechanical Testing

We measured peak ankle power production of the Open Source Leg under continuous myoelectric control in a series of ankle plantar flexion tests off the participant. We created a test apparatus that attached the Open Source Leg to a hinged beam which allowed for free movement of the Open Source Leg while under variable loads. We added loads over the axis of the prosthetic movement to try to replicate participant weight loading. The input signal into our myoelectric controller was a previous recording of raw EMG of an individual without amputation doing a calf raise and followed the same signal processing as the controller methods in Section A. EMG was normalized to 75% of the individual’s maximum voluntary contraction (MVC). Each testing condition consisted of four calf raises while the testing apparatus was loaded in approximately 6.8 kg increments ranging from 40.4 kg to 100.6 kg. On board prosthetics sensors calculated prosthetic power output while a force plate was used to validate test apparatus load. These tests allowed us to verify that the peak ankle power generated by the Open Source Leg running our myoelectric controller would provide suitable ankle power walking with a range of participant weights.

### C. Participants

We recruited five participants with a unilateral transtibial amputation. All participants provided informed and written consent to a protocol previously approved by the University of Florida Institutional Review Board. Before beginning testing, we verified that all participants had the ability to contract their residual muscles volitionally via manual and EMG testing. Table I provides participant physical characteristics. Participants included both vascular and traumatic individuals with amputation, but all participants were capable of walking over 10 minutes continuously. We based the sample size for the study on prior data from our laboratory [22]. That study recruited 5 participants with transtibial amputation and examined gait biomechanics using an experimental pneumatic bionic prosthesis under proportional myoelectric control. The pneumatic prosthesis had to be tethered to a compressed air source, so could only be used on a treadmill in a laboratory. Huang et al. [22] found there were no biomechanical differences between walking with the pneumatic prosthesis and their prescribed passive prosthesis after approximately three hours of training on a treadmill. Based on those a priori statistical predictions and limited recruitment based on clinical availability, we chose to recruit the same number of subjects as in the previous pneumatic study [22].

Participants walked over ground at a self-selected speed while wearing their passive prosthesis and the Open Source Leg under continuous myoelectric control. The prescribed passive prostheses were all energy-storing and return prostheses (Supplementary Table I). Participants wore their passive prosthetic liner and socket with the Open Source Leg. A certified prosthetist determined proper socket alignment for each subject. Due to the height of the Open Source Leg and residual limb length of two participants (2 and 4), the prosthetist fit them with an EVENup (3.175 cm) to correct leg length discrepancies, while others had smaller heel wedges in their intact limb shoe. Additionally, the smallest shoe size compatible with the Open Source Leg was a women’s size 8; therefore, two participants (2 and 4) had a slightly larger shoe on their amputated side.

### D. Sensor Measures

Surface EMG was recorded from the gastrocnemius and tibialis anterior for both legs as well as lower body kinematics and kinetics. For the amputated limb, either the residual medial or lateral gastrocnemius muscle activity was recorded at 450 Hz using the round Neuroline electrodes (Ag/AgCl, 30 × 22 mm) with interelectrode distance approximately 28 mm. Residual limb electrode placement was determined via palpation based on previous literature [35]. The tibialis anterior and lateral and medial gastrocnemius were collected on the intact limb at 450 Hz with round Ag/AgCl Kendall H124SG electrodes (diameter 23 mm) and approximate interelectrode distance of 24 mm. All EMG prep consisted of shaving the area (if hair was present) and cleaning the skin surface with an alcohol wipe. Wires were routed along the legs and secured to reduce motion artifact. OptiTrack Motion Capture system recording at 100 Hz (OptiTrack, Corvallis, OR, USA) collected lower-limb segment kinematics. We used a lower body Rizzoli marker set with iliac crest markers and marker clusters on the thigh and shank. Three AMTI Optima plates (AMTI, Watertown, MA, USA) collected force plate data at 1000 Hz. We placed medial and lateral markers on the powered prosthetic foot approximately at the rotational ankle joint and on the footshell at the height of the intact limb medial and lateral malleolus for the passive prosthesis. Vertical ground reaction forces were also collected for all over ground steps using Novel Loadsol Pressure Insoles sampling at 200 Hz (Novel, Saint Paul, MI, USA). The insole and prosthetic sensor data determined gait events to parse measures such as ankle angle, ankle moment, ankle power as well as muscle activity and sync force plate steps to motion capture measures. Timing gates set up along the middle of the walkway recorded participant walking speed.

### E. Test Protocol

**Fig. 1.**
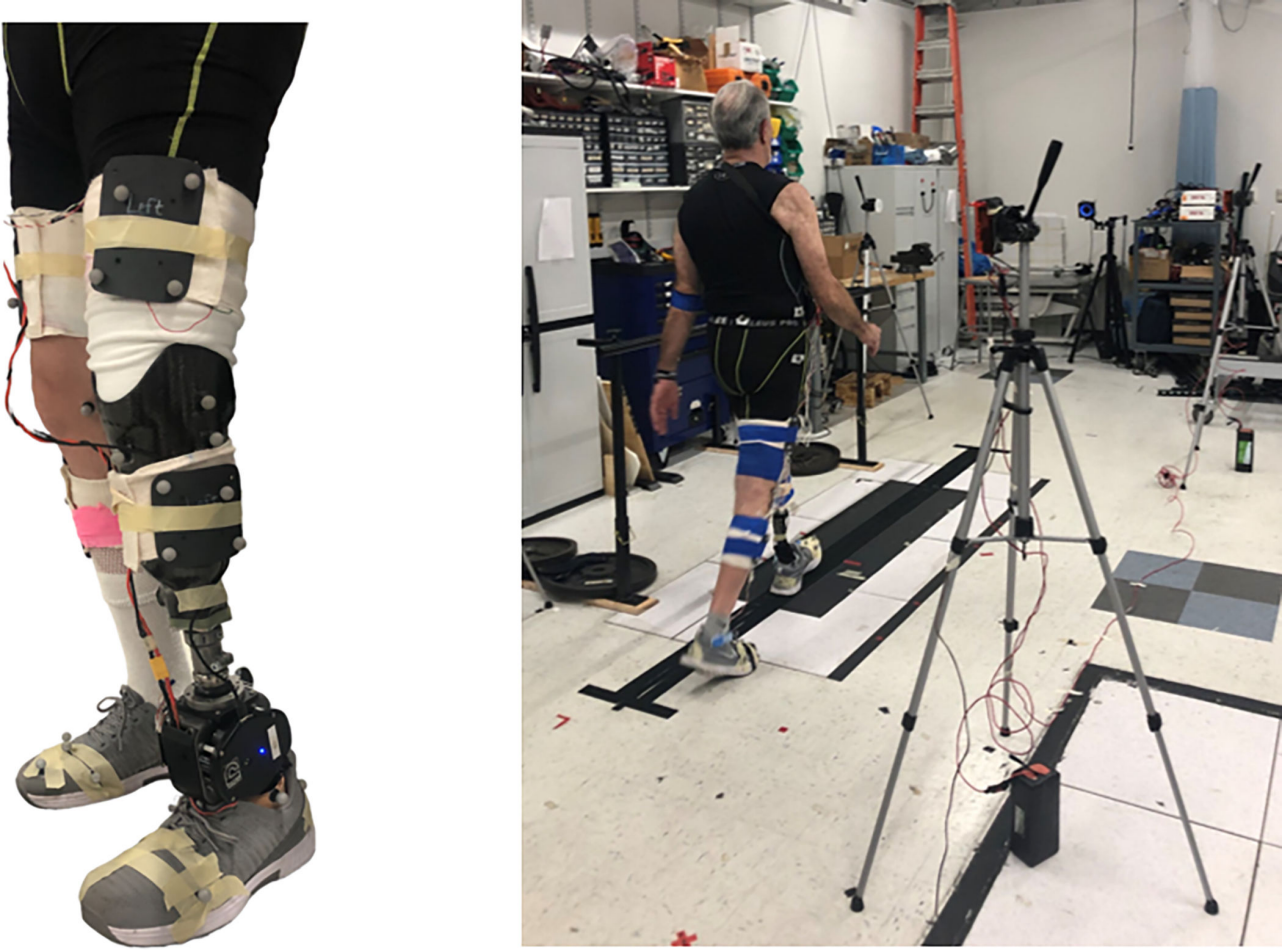
Open Source Leg fitted for a participant (left). Over ground walking testing set up with in ground force plates, parallel bar and timing gates (right).

All participants participated in an over ground walking test trial with data collected both before and after the final training session. For both testing sessions, participants completed over ground walking bouts over a 7.5 m walkway at a self-selected speed until at least six clean force plate strikes were recorded for each leg (one heel strike on a plate) (Fig. 1).

We instructed participants to walk across the pathway without specific descriptors of how to walk. Parallel bars were set up along the force plate area of the walkway to provide support if participants felt unstable. If participants used the parallel bars, the trials were discarded. Participates were given opportunities to rest between trials, if needed, to avoid fatigue. During the initial session, participants completed over ground walking bouts first with their passive prosthesis. Next, a certified prosthetist fit participants with the Open Source Leg and introduced myoelectric control by asking them to push their toes down and tap their toes while seated. Then participants gained insight on how the Open Source Leg behaved when bearing weight by completing calf raises while holding on to the parallel bars. After calf raises, some participants felt comfortable walking along the parallel bars, while others used arm crutches until progressing to walking along the parallel bars. Within 40 minutes of first using myoelectric control, all participants felt comfortable enough to walk unaided. Participants completed their initial over ground prosthetic measurements within their first 20 to 40 minutes of walking with myoelectric control. Participant 1 was an exception to this who had a little over an hour of walking time with myoelectric control before their initial measure due to some prosthetic and controller reliability issues. Within 40 minutes of first using myoelectric control, all participants felt comfortable enough to walk unaided. Participants completed their initial over ground prosthetic measurements within their first 20 to 40 minutes of walking with myoelectric control. Participant 1 was an exception to this who had a little over an hour of walking time with myoelectric control before their initial measure due to some prosthetic and controller reliability issues.

After the initial measurement session, participants returned for multiple training visits to improve their muscle timing while walking with the Open Source Leg under myoelectric control. For training visits, participants were only fitted with EMG sensors on the residual muscle, the Open Source Leg and controller backpack. A maximum voluntary contraction value was collected during each training session for EMG control signal normalization. The EMG threshold was adjusted over the course of training to balance muscle fatigue and prosthetic sensitivity, but still remained between 50–75% of the participant’s maximum voluntary contraction. The number of training visits for each participant and duration of their training is presented in Table II. We found consistent training sessions of one or more per week were needed for participants to maintain their familiarity of walking with myoelectric control and improved their muscle activity adaptation. Training sessions started out as walking along parallel bars over a shorter walkway and progressed to longer hallway walking. Participants received verbal cues to help time muscle activation based on visual inspection of gait and the powered prosthesis, but no visual feedback while walking. Once participants seemed to consistently time their muscle activity and felt stable walking, they progressed to walking on various types of terrains (slopes, curbs, sidewalks/paths) outside around the University of Florida campus. After several training sessions, the prosthetist checked and updated participants’ alignment to account for any changes in gait as they grew more comfortable using myoelectric control.

The final testing session replicated the initial testing session, but the order of walking with their passive prosthesis or the Open Source Leg was randomized. Participant’s walked over the same walkway at a self-selected speed with the Open Source Leg and their passive prosthesis until at least five clean heel strikes for each were recorded on the force plates. Lower limb kinetics, walking speed and EMG for gastrocnemius and tibialis anterior on both legs were collected. We analyzed the walking speed of all participants for all conditions except initial walking with myoelectric control due to faulty data for two participants.

### F. Biomechanical Analysis

For each participant, we analyzed initial and final testing session data. Visual 3D computed inverse dynamics from the last five clean force plate steps on each leg from motion capture data low pass filtered at 10 Hz. The Loadsole pressure insoles and Open Source Leg load cell detected heel strikes and parsed strides for EMG analysis of 20 strides around the force plates from trials with clean force plate strikes. For post processing of EMG measurements, the EMG signals were high-pass filtered (zero-lag second order Butterworth 50 Hz). Then we full wave rectified the high-passed signal and low-pass filtered the full wave rectified signal (zero-lag second order Butterworth 10 Hz). The maximum EMG values from the passive walking condition (initial or final) normalized the intact and prosthetic EMG signals for all conditions. To evaluate EMG activity across conditions, we calculated the root mean squared of the rectified gastrocnemius and tibialis anterior muscles. Ankle moment, mechanical power and mechanical work data were normalized by participant body mass. Peak power was calculated for each participant to account for participant variability in muscle activity timing. We compared residual limb muscle activation patterns, ankle power and ankle work across four walking conditions (passive prosthesis initial session, powered prosthesis initial session, passive prosthesis final session, powered prosthesis final session). We calculated ankle work as the ankle angle integral of the ankle moment curve to understand amount of energy generated by the powered and passive prostheses. Ankle angle, moment, power and work for the powered prosthesis conditions were calculated using Visual 3D. Prosthetic ankle power was calculated with the unified deformable model [36], [37].

To quantify muscle activation profiles, we calculated root mean square (RMS) and variance-to-signal ratio (VRS) of normalized EMG [35]. The root mean square measures for each session (initial and final) were calculated using the last 20 strides with clean force plate strikes and normalized to the root mean square value of each muscle during the passive walking condition for the corresponding visit. The root mean squared values for the gastrocnemius were calculated during stance phase and during swing phase for the tibialis anterior. To analyze repeatability of EMG signals, we calculated a variance-to-signal ratio (VSR) [35]. We used the normalized low-passed filtered EMG data of the last 20 strides for each participant with clean force plate strikes to calculate a variance-to-signal ratio as the sum of the signal variance over the sum of the signal mean for each participant. This was calculated for the gastrocnemius and tibialis anterior muscles for both limbs.

### G. Preference Questionnaire

After completing the final measurement session, participants completed a preference questionnaire [38]. The preference questionnaire consisted of one question where participants were asked which prosthesis they preferred walking with over ground. The question was on a 100 mm visual analog scale from passive prosthesis (0) to myoelectric control (10).

### H. Statistical Analysis

Due to the within subjects research design with individuals serving as their own control, a two-factor repeated measure design was used to compare prosthesis type (prescribed passive versus powered) by training time (initial and final testing session) and interaction effects (prosthesis by time). Analyses for peak power, ankle work and EMG variance to signal noise ratio were performed using a two-factor ANOVA with SPSS version 29.0 (IBM SPSS Statistics for Windows, Armonk, NY: IBM Corp). EMG RMS and walking speed analysis were computed with a repeated measure ANOVA. An alpha level of 0.05 was used to denote statistical significance. When a significant main effect (p < 0.05) was found, a Bonferroni adjustment (for multiple comparisons) was used to determine which means were significantly different from each other. We assessed if prosthesis preference was significantly different than 50 mm (no preference) using a one sample t-test with a significance level set at p < 0.05. If the data in any single measurement violated assumptions of normality based on Shapiro-Wilk’s test, a Friedman’s test was used instead of ANOVA.

We report partial eta^2^ or Hedges’ g for dependent variables of interest. Partial eta-squared $\left(\eta_{p}^{2}\right)$ reported in SPSS express the amount of variance accounted for by one or more independent variables, and are generally used in conjunction with ANOVA with repeated measures [39]. Hedges’g effect sizes and 95% confidence intervals (CI) were calculated using Comprehensive Meta-Analysis Version 3.3070 (Biostat, Englewood NJ). Similar to Cohen’s d (but corrected for small sample size), Hedges’ g effect sizes > 0.2 were considered small, > 0.5 moderate, and > 0.8 large magnitudes of effect. For partial eta^2^ effect sizes > 0.01 were considered small, > 0.06 moderate, and > 0.14 large magnitudes of effect.

## III. Results

### A. Mechanical Testing

**Fig. 2.**
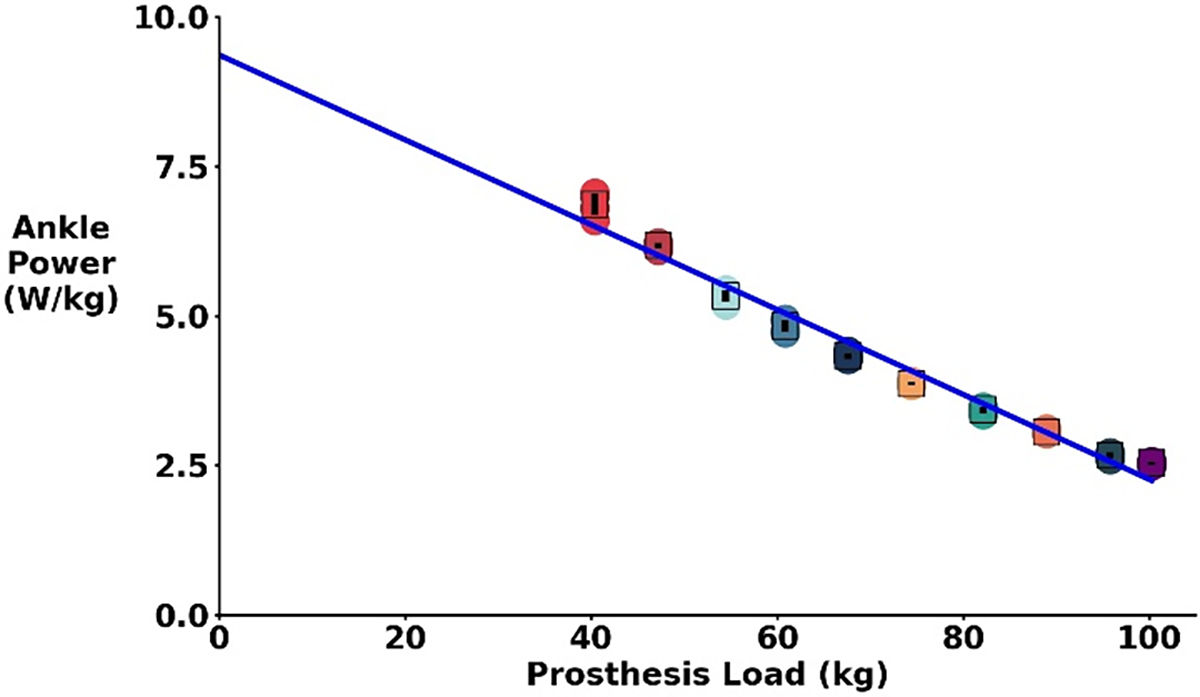
Normalized peak ankle power generation of the Open Source Leg mounted on testing apparatus under continuous myoelectric control under 10 different loading conditions. The prosthetic control input was an averaged EMG signal from a non-amputee performing calf raises. The power value is an average of four calf raises per condition. Peak power is on the Y axis normalized by body mass while the X axis is the range of prosthetic loads tested approximately from 40.4 to 100.6 kg in approximate 6.8kg (15 pound) increments. (Linear trend line R^2^: 0.97). For all weight conditions, accounting for our participant’s weights, the Open Source Leg and myoelectric controller was able to generate at least 2.5 W/kg of peak ankle power.

For all test apparatus loading conditions, the prosthesis was able to produce peak ankle power values characteristic of intact walking biomechanics. The prosthesis generated over 2.5 W/kg of peak ankle power for all loading trials (Fig. 2). The lowest peak power production was for the heaviest condition at 100.6 kg at 2.55 ± 0.03 W/kg while the highest peak power was at the 40.3 kg condition at 6.87 ± 0.18 W/kg. Based on these results, participants weighing 100.6 kg and under should be able to achieve a peak ankle power of at least 2.5 W/kg.

### B. Walking Speed

Walking speeds were similar across conditions and with both prostheses. The average walking speeds for all participants for the initial passive, final passive and final myoelectric walking conditions were 1.1 m/s respectively. There were no significant differences in walking speed between the initial passive, final passive and final myoelectric walking conditions (p = 0.916). The initial powered walking speed for the three available participants was 0.9 m/s. The average walking speed for participants we had values on are in Supplementary Table II.

### C. Peak Ankle Power

**Fig. 3.**
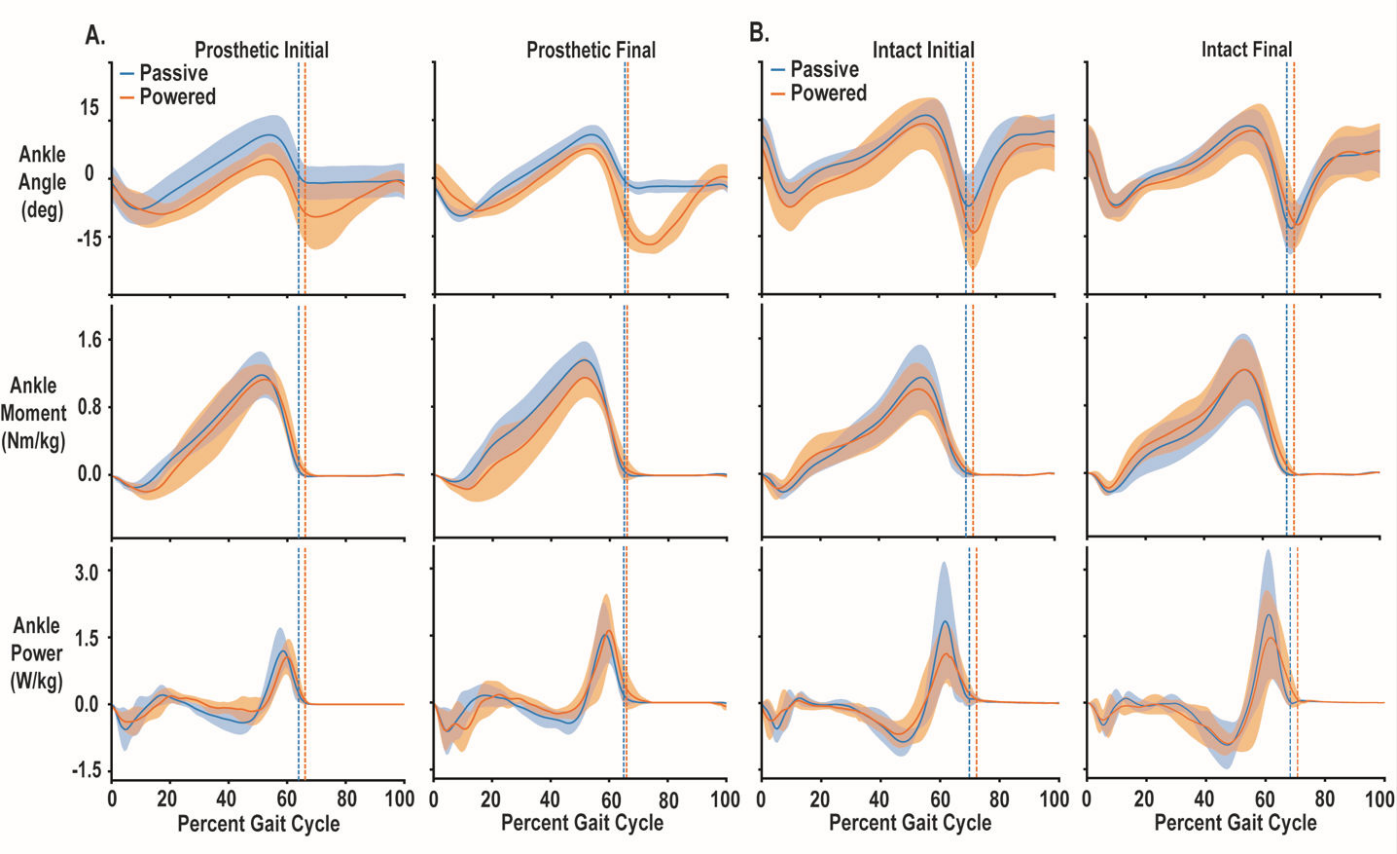
Time series ankle biomechanics measures for all participants comparing walking with their passive prosthesis during the initial visit and powered prosthesis during the final visit. Measures include ankle angle where dorsiflexion is positive and plantar flexion is negative, ankle moment, ankle power and vertical ground reaction force for the prosthetic (A) and intact (B) side. Blue represents passive prosthesis while orange represents continuous myoelectric control. The vertical dashed lines represent toe off and shaded regions represent ± 1 standard deviation. The top group of plots display prosthetic limb measures while the bottom are the intact limb measures. R ^2^ values for subject power ankle power by passive ankle power were 0.54±0.17. There were no significant differences in peak ankle power between the powered (2.10±0.71 W/kg) and passive (1.75±0.70) prostheses. However, during the final testing session four out of five participants generated more ankle power with the powered prosthesis compared to their passive devices.

**Fig. 4.**
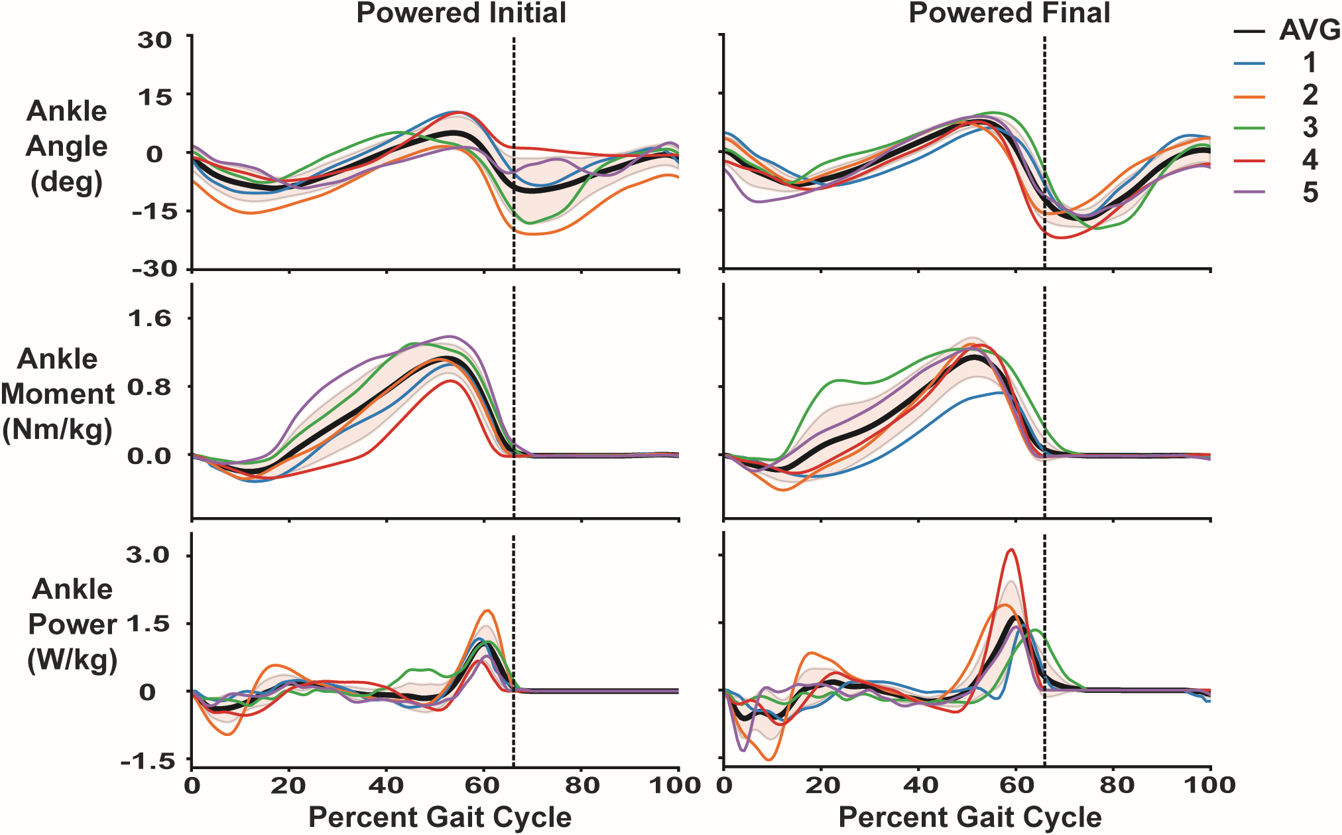
Participant group average and individual average for ankle angle, normalized ankle moment and ankle power for the initial and final powered prosthesis walking conditions normalized across a gait cycle. These values were calculated based on the final five clean force plate strikes for each participant. The black line represents the group participant average while the colored lines represent each individual participant’s average. The shaded grey region is ± 1 standard deviation. The vertical black dashed line indicates toe off for the group participant average. Participants on average increased their peak ankle power walking with the powered prosthesis by 0.80±0.31 W/kg between the initial and final testing session.

All but one participant for the powered prosthesis and all participants for the passive prosthesis increased their peak ankle power between testing session (Fig. 3A; Fig. 4). Two factor repeated measures ANOVA for ankle power indicated no differences based on prosthesis (p = 0.704, $\eta_{p}^{2}=0.040$), prosthesis by time interaction (p = 0.348, $\eta_{p}^{2}=0.220$), or familiarization time (initial vs final visit) (p = 0.095, $\eta_{p}^{2}=0.542$). The mean (± SD) difference in peak power was 0.80 ± 1.02 W/kg and 0.39 ± 0.31 W/kg, for myoelectric controlled and passive prostheses, respectively. Effect sizes (g) for increases in peak ankle power (between initial and final tests) for powered (g = 1.11, 95% CI: −0.61–2.820; p = 0.206) was not significant, while passive (g = 0.46, 95% CI: 0.12–0.79; p < 0.008) prostheses was significant. The mean (± SD) difference in peak power between prostheses during the final visit was 0.36 ± 1.07 W/kg, favoring the myoelectric controlled prosthesis. This was a non-significant difference (p = 0.48) with a small effect (g = 0.41). However, after practicing, four of the five participants were able to exhibit greater ankle power on myoelectric controlled prosthesis by at least 0.5 W/kg (mean difference = 0.8 W/kg, g = 0.34, p = 0.002) compared to their passive prosthesis. The group time series average for these measures for visit (initial and final) and prosthesis (passive and powered) are displayed in Fig. 3A for the prosthetic limb and 3B for the intact limb. The time series data for ankle angle, ankle moment and ankle power for each individual participant comparing the initial powered and final powered conditions are presented in Fig. 4. Supplementary Tables III and IV contain the intact and residual peak ankle power measures for all participants.

### D. Prosthetic Ankle Work

**Fig. 5.**
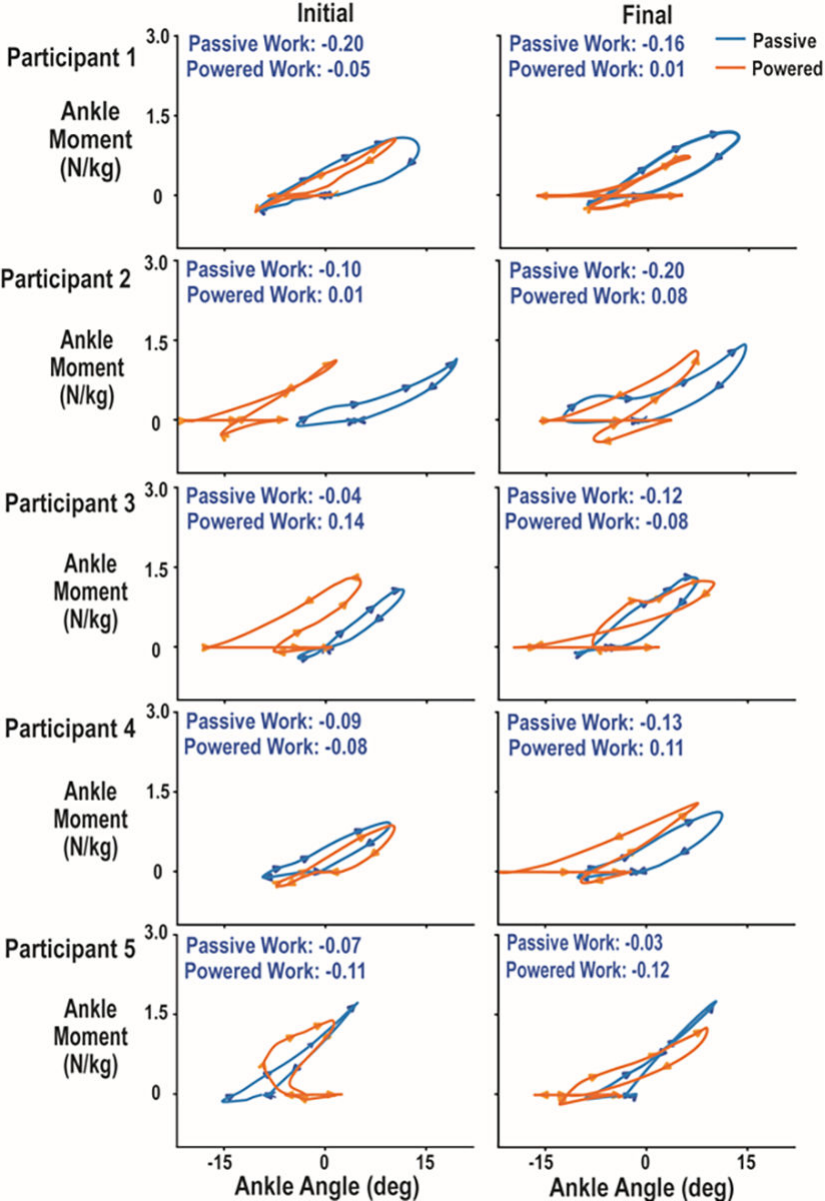
Individual prosthetic ankle work (J/kg) loops over the five step averaged gait cycle calculated as the integral of the ankle moment over change in ankle angle. Blue lines represent the participants’ passive prosthesis and orange lines are the powered prosthesis under myoelectric control. The arrows in each line indicate the direction the curve moves over a step. Negative ankle angle values correspond to plantar flexion while positive values are dorsiflexion. The right column of plots is for the initial testing session while the left column corresponds for the final testing session. Three out of five participates produces positive net work with the powered prosthesis. Participants on average generated greater work with the powered prosthesis than their passive prosthesis at the final session, although not statistically significant.

Three out of five participants generated net positive work walking with the powered prosthesis by the final visit assessment. Mean difference between powered and passive prostheses was 0.13 ± 0.15 J/kg. Fig. 5 and Supplementary Table V provides individual subject work loop curves and values for each prosthesis for the initial and final visits. A two factor repeated measures ANOVA found no differences between prostheses (p = 0.080, $\eta_{p}^{2}=0.577$), prosthesis by time interaction (p = 0.538, $\eta_{p}^{2}=0.101$) or familiarization time (p = 0.905, $\eta_{p}^{2}=0.004$). Effect size for the increased ankle work generated with the final powered compared to final passive trial was large (g = 1.26), but there was not a statistically significant difference for the five subjects (p = 0.208).

### E. EMG RMS and VSR

**Fig. 6.**
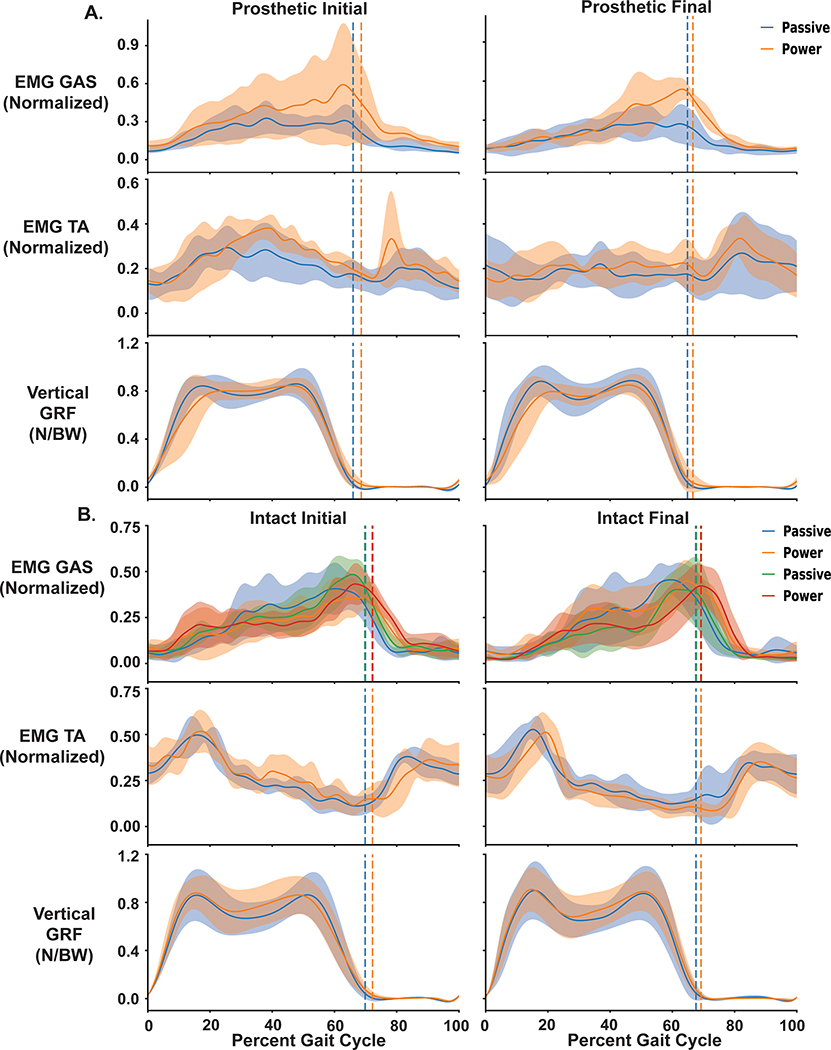
Stride normalized EMG of the tibialis anterior and gastrocnemius muscles on the prosthetic (A) and intact (B) limb for participants walking with the powered and their passive prosthesis during their initial (Left) and final (Right) visit post training. For the intact limb the medial and lateral gastrocnemius were recorded. Measures include normalized low passed muscle activity and normalized vertical ground reaction force. For the ground reaction forces, tibialis anterior, and prosthetic gastrocnemius measures, blue represents the participant’s passive prosthesis while orange is the powered prosthesis under myoelectric control. For the intact limb gastrocnemius muscles, blue and green represent the passive conditions and orange and red are the powered conditions for the medial and lateral gastrocnemius, respectively. Heel strike represents 0% on the x axis. The vertical bar represents toe off and shaded regions represent ± 1 standard deviation. Participants seem to reduce the variability of their residual gastrocnemius muscle activity between initial and final testing sessions, while the intact limb muscle activity remains similar between initial and final sessions.

**Fig. 7.**
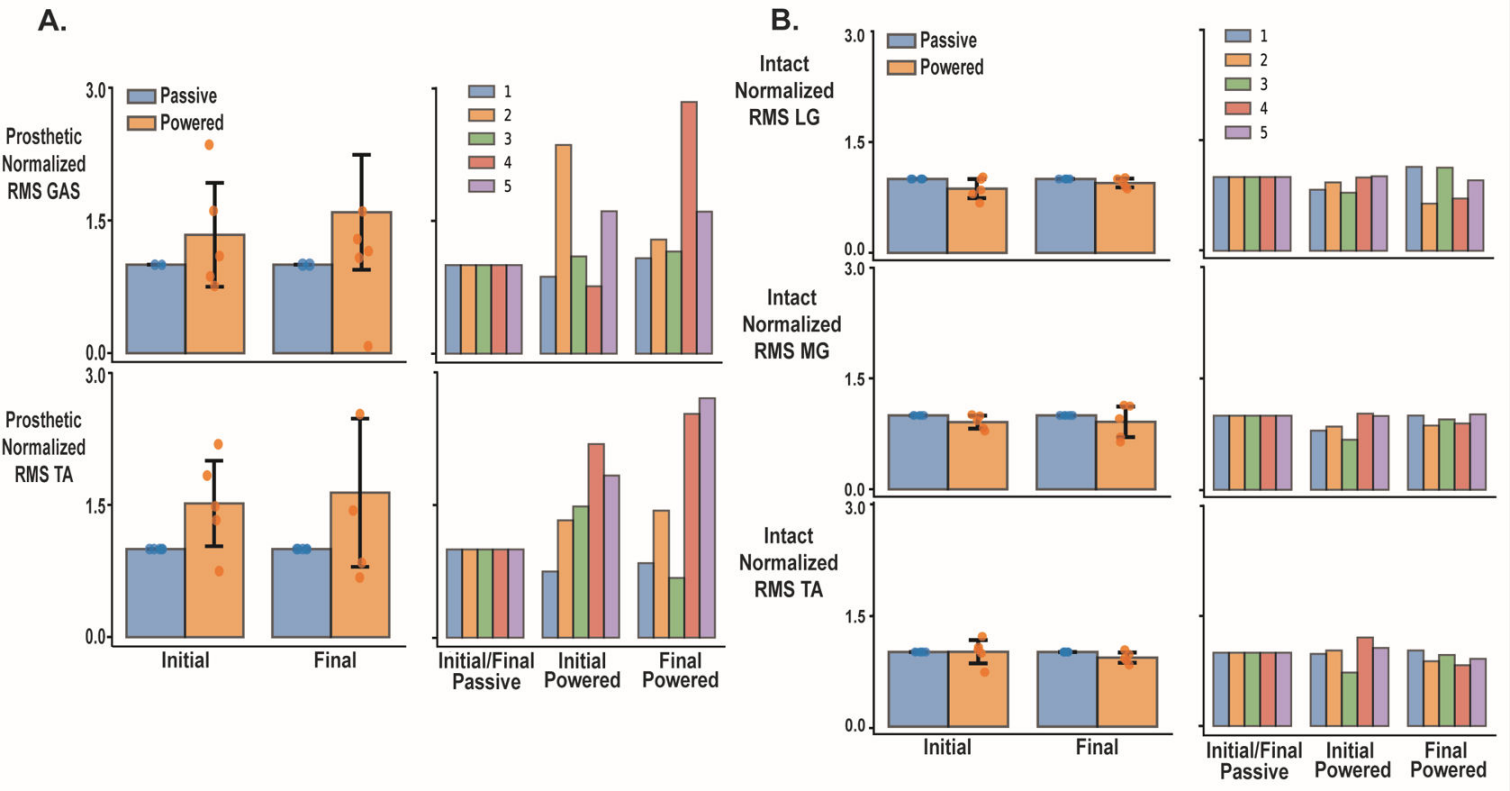
The root mean squared (RMS) EMG of the residual gastrocnemius and tibialis anterior muscles for all participants and each individual participant (A) and intact limb lateral (LG) and medial (MG) gastrocnemius and tibialias anterior muscles (B). Error bars are ± 1 standard deviation and individual data points are represented on the group average plots. Root mean squared values are normalized to the passive prosthetic walking for the initial or final session. For group average plots (right), EMG RMS is grouped by initial and final visit and blue represents passive and orange the powered walking conditions. The individual plots (left) are grouped by prosthetic walking condition (Initial/Final Passive, Initial Power and Final Power). The passive walking conditions are grouped together since they serve as the normalizing conditions. Over all, there were no significant differences in RMS EMG across conditions, however there was a large variability between individuals.

**Fig. 8.**
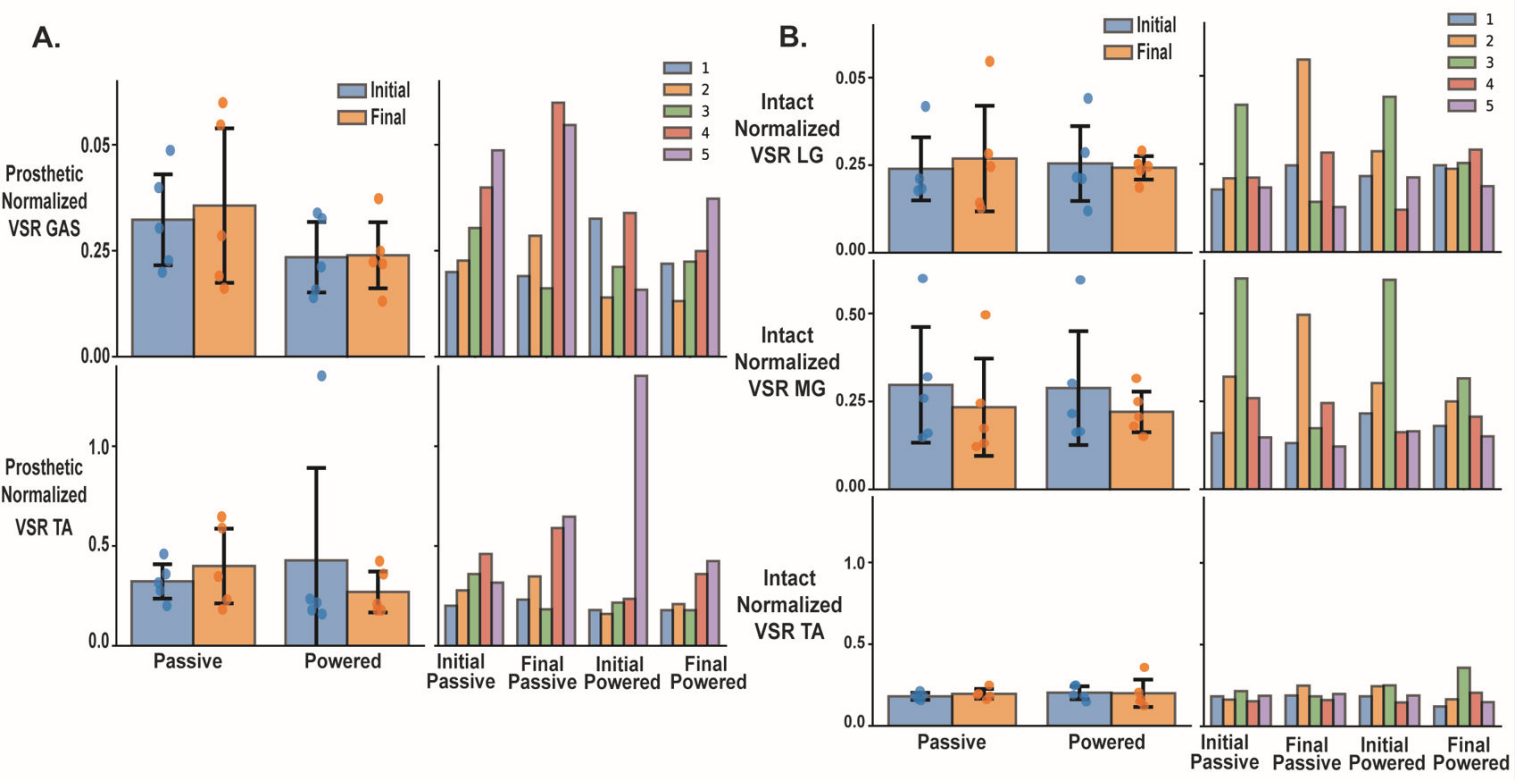
The Variance-to-Signal Ratio (VSR) for EMG of the residual gastrocnemius and tibialis anterior muscles for all participants and each individual participant (A) and each individual participant (B) and intact limb lateral (LG) and medial (MG) gastrocnemius and tibialis anterior muscles (right). Error bars are ± 1 standard deviation and individual data points are represented on the group average plots and grouped by prosthesis walking condition (passive and powered). Blue represents VSR measure for initial visit while orange is for the final visit. For the individual group plots VSR measures are grouped by each walking condition and visit (Initial Passive, Final Passive, Initial Power, and Final Power). Over all, there were no significant differences in VSR EMG across conditions, however there was a large variability between individuals.

We found no significant differences in EMG amplitudes or variability metrics between testing session or prosthesis. The time series linear envelope and rectified EMG for all session and prosthetic walking conditions are shown in Fig. 6A for the prosthetic and Fig. 6B for the intact limb. Fig. 7 displays group and individual RMS values for intact and residual gastrocnemius and tibialis anterior muscles. Residual RMS values for initial and final passive prosthesis initial and final powered walking were not significant for the gastrocnemius (p = 0.218, $\eta_{p}^{2}=0.638$), or tibialis anterior muscles respectfully (Fig. 7A) (p = 0.316, $\eta_{p}^{2}=0.536$). The effect size for the difference between RMS measures for passive and powered prosthesis for the residual gastrocnemius muscle at the initial visit was significant (g = 0.447, p = 0.046) but not at the final visit (ES = 0.38; 95%CI −0.05 – 0.81; p = 0.084). Similarly, we did not see significant differences in RMS EMG for the intact limb medial (p = 0.193, $\eta^{2}=0.666$) and lateral (p = 0.108, $\eta_{p}^{2}=0.773$) (Fig 7B) gastrocnemius and tibialis anterior (p = 0.218, $\eta_{p}^{2}=0.638$) muscles across prosthetic walking conditions. For VSR, there was no significant difference comparing passive and powered prosthesis for each testing session for the residual tibialis anterior (p = 0.145), or intact medial gastrocnemius (p = 0.472). VSR values for the residual shank muscles trended towards more significance than the intact shank muscles (Fig. 8A). The residual tibialis anterior and intact lateral gastrocnemius violated normality and were analyzed with a Friedman’s test. We saw no significant difference within the three muscle groups (residual gastrocnemius, intact medial gastrocnemius, intact tibialis anterior) due to prosthesis type (p = 0.165, $\eta_{p}^{2}=0.419$); p = 0.785, $\eta_{p}^{2}=0.021$; p = 0.629, $\eta_{p}^{2}=0.064$), across visit (p = 0.641, $\eta_{p}^{2}=0.060$; p = 0.423, $\eta_{p}^{2}=0.166$; p = 0.661, $\eta_{p}^{2}=0.053$), or the interaction between prosthesis and visit respectively (p = 0.748, $\eta_{p}^{2}=0.029$; p = 0.953, $\eta_{p}^{2}=0.001$; p = 0.740, $\eta_{p}^{2}=0.31$) (Fig. 8B). For EMG time series data (Fig. 6), we did not use statistical parametric mapping due to the low power of our study [40]. However, Fig. 6 shows how the variance of the residual gastrocnemius muscle decreases from initial to final powered walking. Additionally, there seems to be a larger magnitude of the normalized residual gastrocnemius EMG when walking with myoelectric control compared to participant’s passive prosthesis at toe off for the final testing session.

### F. Preference Questionnaire

Participants preferred the powered prosthesis compared to the passive prosthesis. Participants preferred walking with the powered prosthesis under continuous proportional myoelectric control compared to their passive prosthesis (preference score of 7.48 ± 0.976, g = 2.028), which was significantly different from no preference (p = 0.005).

## IV. Discussion

Our main finding was individuals with transtibial amputation were able to walk with the powered prosthesis under myoelectric control with about five hours of practice and even preferred it to their passive prosthesis. We found that individuals with amputations due to vascular and trauma could modify their residual muscle activity to more reliably generate ankle plantar flexion and ankle power with our prosthesis under myoelectric control (Fig. 3A). The unique features of our study were: 1) training occurred during over ground walking inside at self-selected pace instead of a treadmill and, 2) our prosthesis was portable with an electromechanical actuator instead of pneumatic actuator. Our results suggest training conditions and actuator type do not inhibit individuals with amputation from learning to modify their muscle activity to walk with a prosthesis under proportional myoelectric control.

Based on controller and prosthetic mechanical testing, all participants theoretically could have achieved larger peak ankle power while walking with the powered prosthesis under continuous myoelectric control. While power output decreased with increasing prosthetic load (Fig. 2), based on mechanical testing the Open Source Leg under myoelectric control should have been able provide at least 2.5 W/kg of peak power for all the walking conditions. There could be several possible explanations for the relatively lower mechanical power output during walking. We did not provide any visual feedback of muscle activity during training. Based on the results of Huang et al. [22], providing participants with visual feedback and asking them to increase ankle mechanical power leads to larger peak ankle power compared to walking with a prescribed passive prosthesis. Four out of the five participants experienced multiple belt slips during training sessions. A belt slip did not cause any instability while walking, but did produce a jarring noise that the participants noticed. Open Source Leg belt slips have also been reported by Posh et al. [20]. A second generation Open Source Leg has been developed which could reduce belt slip issues. Participant 4, did not experience belt slips, had the lightest mass of all participants (64 kg compared to ~ 90 kg for all others), and performed exceptionally with our powered prosthesis. The increased power change normalized to body mass from initial to final session in participant 4 was 2.59 W/kg while all other subjects combined averaged 0.36 W/kg. It is possible that there is an optimal participant mass for our myoelectric controller on the Open Source Leg. Finally, using EMG as a control signal leads to high variability in powered prosthetic power output which is a plus and minus of direct myoelectric control. With additional practice, participants may have reduced the variability of their peak power output. No studies have investigated long term use of lower limb direct myoelectric control adaptation.

Training resulted in improvements in mechanical power during walking for all participants except participant 5. Participant 5 had the greatest body mass and peak power generation with their passive device (2.93 ± 0.48 W/kg) compared to all other participants combined (1.45 ± 0.26 W/kg). The passive prostheses for all these participants were energy restoring feet, in contrast to participant 5 who used an Endolite Blade foot which could be closer to a cross over foot combining features of an energy restoring foot and a running blade [41]. We believe the features of a cross over foot, could explain the large passive peak ankle power generation of participant 5 compared to all other participants [42], [43]. Additionally, participant 5 generated the smallest peak ankle power with the powered prosthesis 1.52 ± 0.77 W/kg compared to 2.25 ± 0.73 W/kg for all other participants combined. This could be due to the larger body mass and/or time since amputation (38.5 years); however, further analysis would be needed. When removing participant 5 as an outlier, a two factor repeated measures ANOVA for peak ankle power indicated no difference for time (initial vs final visit) (p = 0.179, $\eta_{p}^{2}=0.504$) or prosthesis by time interaction (p = 0.322, $\eta_{p}^{2}=0.332$), but a significant difference in prosthesis (p < 0.001, $\eta_{p}^{2}=0.993$). Post hoc tests indicated significant increase (p = 0.045) in final peak ankle power for the powered prosthesis. It is unclear whether participant 5 is a true outlier or representative of the variability that is inherent in the population of individuals with transtibial amputation.

There were changes in mechanical power and work produced across both powered and passive prostheses. We did see on average a smaller change in passive prosthetic peak power between testing sessions, but it was significant. The changes in ankle power and work generation could potentially be from participants being more comfortable walking in a lab set up wearing sensors and demonstrated their more natural walking gait. Additionally, practicing with the powered prosthesis could have altered how participants walk with their passive device. Additional studies would be needed to understand this further.

For walking speed, it has been suggested that a minimum clinically important difference is 0.21 m/s [44]. Based on previous work [45], an increase in walking speed of 0.21 m/s corresponds to a change in ankle power of 0.82 W/kg with an estimated effect size of d = 1.36. The differences we found in the powered prosthesis with practice walking are close to this estimated clinically meaningful difference, but future studies would need to confirm this estimate.

There were no significant differences in muscle activity measures between walking with the passive and powered prostheses, but there were tendencies to shift the magnitude and timing of residual muscle activity in the practice time provided in the study. Comparing the initial and final gastrocnemius muscle activity during walking with the powered prosthesis, participants reduced their muscle activity in early stance to time the peak of their muscle activity near toe off (Fig. 6). Between initial and final powered prosthesis walking, there were trends for reduced variability in muscle activation patterns that appear in VSR. However, the muscle activity for the intact limb did not seem to change during any of our initial and final walking conditions.

Participants significantly preferred walking with the powered prosthesis under myoelectric control compared to their own passive prosthesis. This difference in subjective preference occurred even though there was no significant difference in peak power outputs between the two prostheses. This finding suggests that peak power generation is not the main performance metric of users, in line with previous findings [46]. More work is needed to understand if the preference of myoelectric control after multiple walking sessions stems from the increased range of motion that would be provided by any powered prosthesis or the volitional nature of the control strategy.

There were some limitations to this initial pilot study. We did not control the over ground walking speed, but participants did not vary their walking speed between prostheses very much. We did not control the total prosthetic training time or number of sessions. There was a relatively small amount of training provided and the main point was to make sure that the users felt comfortable and safe with the powered prosthesis [47], [48], [49]. Given the outcome in participant preference, the amount of time was sufficient for the goal. Interestingly, some participants became fatigued activating their residual muscles to control the powered prosthesis because they had not consistently activated them since prior to amputation. This finding was similar to the prior study with a pneumatic powered prosthesis [22]. The time since amputation for our participants ranged from 0.4 to 38.5 years. A future study that follows participants over a longer training period could determine if the time since amputation is an important factor in proportional myoelectric control of powered lower limb prostheses. Lastly, use of a visual analog scale [38] could be open to bias by participants.

## V. Conclusion

We demonstrated that individuals with transtibial amputation could learn to walk with an untethered bionic ankle prosthesis under continuous proportional myoelectric control. After multiple walking sessions with the powered prosthesis, most participants increased ankle work and peak ankle power compared to their own passive device. Based on mechanical testing of the prosthesis on a test apparatus, participants’ activated the prosthesis so that the average peak power was well below the device’s peak power output capacity. Participants preferred walking with the powered prosthesis under myoelectric control compared to their passive prosthesis. This suggests that direct volitional control could influence user prosthetic satisfaction in future devices. While testing additional participants with the current myoelectric controlled prosthesis would add statistical power to the comparison between the prostheses, our findings highlight additional training methods (e.g. such as visual feedback) may be needed to elicit high mechanical power output from a myoelectric ankle prosthesis. Participants walked with about half the peak power the prosthesis was capable of.

## Supplementary Material

supp1-3440257

## Figures and Tables

**TABLE I:** Unilateral Transtibial Amputee Participant Details

Participant Number	Sex	Age (years)	Height (m)	Weight (kg)	Years Post Surgery	Reason for Amputation
1	M	54	1.75	86	1.5	Vascular
2	F	48	1.73	87	2	Vascular
3	M	66	1.8	92	0.4	Vascular
4	F	17	1.63	64	2	Trauma
5	M	65	1.95	93	38.5	Trauma

**TABLE II:** Participant Walking Totals During Training Visits

Participant Number	Number of Training Visits	Total Prosthesis Walking Time (hrs)	Visit Walking Time/Min (mean)	Training Duration (months)
1	5	2.9	26	1.3
2	8	5.3	40	3
3	5	4	48	1
4	7	5.3	46	2.2
5	9	6.2	43	1.3
MEAN ± SD	6.8 ± 1.8	4.7 ± 1.3	40.6 ± 8.7	1.8 ± 0.8
