# Supplementary material for: Walking Ankle Biomechanics of Individuals With Transtibial Amputations Using a Prescribed Prosthesis and a Portable Bionic Prosthesis Under Myoelectric Control
Source: IEEE Trans Neural Syst Rehabil Eng. Author manuscript; Available in PMC 2024 Nov 13. (PMC11559236; doi:10.1109/TNSRE.2024.3440257)
Supplement: supp1-3440257 [file NIHMS2019084-supplement-supp1-3440257.docx]

# Supplementary Material

TABLE I: Participant Prosthetic Componentry details. The height of the even up was 3.175 cm and average height of heel wedges was 1 cm.

| Participant Number | Suspension Type | Prosthetic Liner | Prescribed Prosthesis | Shoe Size | OSL Setup |
| --- | --- | --- | --- | --- | --- |
| 1 | Pin | Gel | Kinterra Proteor | 10 M | No additional offsets |
| 2 | Suction | Gel | Ossur Talux | 6 W | EVENup |
| 3 | Pin | Gel | Ottobock Trias | 10 M | No additional offsets |
| 4 | Pin | Gel | Ossur Vari-Flex | 5 W | EVENup |
| 5 | Suction | Gel | Elite Blade Endolite | 13 M | Heel Wedge |

TABLE II: Prosthetic Walking speed for each participant for their initial and final visits after training for each prosthesis type. We were unable to measure initial powered prosthesis walking for participants 2 and 3 because of equipment malfunction. Peak ankle power is a five step average calculated from over ground force plate and motion capture data. Values include ± 1 standard deviation.

| Participant Number | Initial Session Passive Prosthesis (m/s) | Final Session Passive Prosthesis (m/s) | Initial Session Powered Prosthesis (m/s) | Final Session Powered Prosthesis (m/s) |
| --- | --- | --- | --- | --- |
| 1 | 1.1 | 1.0 | 1.0 | 1.1 |
| 2 | 1.1 | 1.1 | X | 1.2 |
| 3 | 0.9 | 0.9 | X | 0.9 |
| 4 | 0.9 | 1.1 | 1.0 | 1.1 |
| 5 | 1.4 | 1.3 | 0.8 | 1.1 |
| MEAN ± SD | 1.1 ± 0.2 | 1.1 ± 0.1 | 0.9 ± 0.1 | 1.1 ± 0.1 |

TABLE III: Prosthetic ankle peak power for each participant for their initial and final visits after training for each prosthesis type. Peak ankle power is a five step average calculated from over ground force plate and motion capture data. Values include ± 1 standard deviation.

| Participant Number | Initial Session Passive Prosthesis | Final Session Passive Prosthesis | Initial Session Powered Prosthesis | Final Session Powered Prosthesis |
| --- | --- | --- | --- | --- |
| 1 | 0.80 ± 0.16 | 1.40 ± 0.19 | 1.35 ± 0.76 | 1.96 ± 0.76 |
| 2 | 1.43 ± 0.11 | 1.42 ± 0.20 | 1.94 ± 0.57 | 1.99 ± 0.31 |
| 3 | 1.05 ± 0.19 | 1.16 ± 0.30 | 1.39 ± 0.39 | 1.72 ± 0.53 |
| 4 | 1.16 ± 0.30 | 1.81 ± 0.39 | 0.74 ± 0.16 | 3.32 ± 0.84 |
| 5 | 2.33 ± 0.25 | 2.93 ± 0.48 | 1.08 ± 0.49 | 1.52 ± 0.77 |
| MEAN ± SD | 1.35 ± 0.59 | 1.75 ± 0.70 | 1.30 ± 0.44 | 2.10 ± .71 |

TABLE IV: Intact ankle peak power for each participant for their initial and final visits after training for each prosthesis type. Peak ankle power is a five step average calculated from over ground force plate and motion capture data. Values include ± 1 standard deviation.

| Participant Number | Initial Session Passive Prosthesis | Final Session Passive Prosthesis | Initial Session Powered Prosthesis | Final Session Powered Prosthesis |
| --- | --- | --- | --- | --- |
| 1 | 1.13 ± 0.14 | 1.26 ± 0.24 | 1.32 ± 0.31 | 1.32 ± 0.32 |
| 2 | 0.47 ± 0.09 | 0.58 ± 0.17 | 0.45 ± 0.13 | 0.47 ± 0.06 |
| 3 | 1.72 ± 0.30 | 2.03 ± 0.47 | 1.23 ± 0.16 | 1.86 ± 0.38 |
| 4 | 3.37 ± 0.46 | 3.86 ± 0.62 | 2.58 ± 0.18 | 3.02 ± 0.17 |
| 5 | 4.16 ± 0.10 | 3.93 ± 0.31 | 1.80 ± 0.81 | 3.46 ± 0.44 |
| MEAN ± SD | 2.17 ± 1.55 | 2.33 ± 1.52 | 0.78 ± 0.78 | 2.03 ± 1.22 |

TABLE V: Net prosthetic ankle work for each participant for their initial and final visits after training for each prosthesis type. Peak ankle power is a five step average calculated from over ground force plate and motion capture data. Values include ± 1 standard deviation.

| Participant Number | Initial Session Passive Prosthesis | Final Session Passive Prosthesis | Initial Session Powered Prosthesis | Final Session Powered Prosthesis |
| --- | --- | --- | --- | --- |
| 1 | -0.2 ± 0.02 | -0.16 ± 0.01 | -0.05 ± 0.05 | 0.01 ± 0.04 |
| 2 | -0.10 ± 0.02 | -0.20 ± 0.04 | 0.01 ± 0.07 | 0.08 ± 0.05 |
| 3 | -0.04 ± 0.02 | -0.12 ± .02 | 0.14 ± 0.01 | -0.08 ± 0.06 |
| 4 | -0.09 ± 0.01 | -0.13 ± 0.02 | -0.08 ± 0.01 | 0.11 ± 0.08 |
| 5 | -0.07 ± 0.04 | -0.03 ± 0.04 | -0.11 ± 0.08 | -0.12 ± 0.10 |
| MEAN ± SD | -0.10 ± 0.06 | -0.13- ± 0.07 | -0.02 ± 0.11 | -0.01 ± 0.10 |

S1. Participant Controller and Impedance Parameters

For the impedance controller we used the default function from the Dephy Actpack library and the default kp (40), ki (400) values and stayed within their suggested range of Stiffness (K): [0 2000] and Damping (B): [0 20000] values (<https://dephy.com/start/#control-gains>). The stiffness and damping values correspond to the Dephy stiffness and parameter values for their low level controller. The set angle in degrees was mapped to motor ticks for the on board encoder inside the Dephy Actpack. We had three impedance stiffness and damping changes for early stance/swing, mid-stance and late stance. For swing and early to mid-stance, the stiffness (K)was set to 2000 and damping was 5500. Once participants began to load the prosthesis we reduced the stiffness and damping parameters to more closely mimic impedances of a biological ankle. This parameter transition was determined by the sagittal plane moment of the Open Source Leg onboard load cell (threshold value < [-15 to -10] Nm). We again updated the stiffness and damping parameters based on participant loading determined by the sagittal plane moment of the Open Source Leg onboard load cell (threshold value > [45 to 50] Nm). When the prosthesis load was greater than 65% of the participant’s body weight combined with the sagittal plane moment we determined the participant was transitioning to plantarflexion push off. The stiffness was the same as early stance/swing K=2000, but the damping parameter was scaled based on the participant’s weight. The damping ranged between 5777 and 7722 proportional to the participants’ weight ranging from 140 to 210 pounds. Once the load of the prosthesis was less than 30% of the participant’s body weight the stiffness and damping parameters transitioned back to early stance/swing values. The average delay for the controller with EMG processing was 122 ms.
